# Recent Advances of WEE1 Inhibitors and Statins in Cancers With p53 Mutations

**DOI:** 10.3389/fmed.2021.737951

**Published:** 2021-10-04

**Authors:** Xiangbing Meng, Jason Z. Gao, Sean Michael T. Gomendoza, John W. Li, Shujie Yang

**Affiliations:** ^1^Department of Pathology, The University of Iowa, Iowa City, IA, United States; ^2^Holden Comprehensive Cancer Center, Carver College of Medicine, University of Iowa, Iowa City, IA, United States; ^3^Department of Human and Evolutionary Biology, University of Southern California, Los Angeles, CA, United States

**Keywords:** p53, Wee1, AZD1775, adavosertib, statins, ZN-c3

## Abstract

p53 is among the most frequently mutated tumor suppressor genes given its prevalence in >50% of all human cancers. One critical tumor suppression function of p53 is to regulate transcription of downstream genes and maintain genomic stability by inducing the G1/S checkpoint in response to DNA damage. Tumor cells lacking functional p53 are defective in the G1/S checkpoint and become highly dependent on the G2/M checkpoint to maintain genomic stability and are consequently vulnerable to Wee1 inhibitors, which override the cell cycle G2/M checkpoint and induce cell death through mitotic catastrophe. In addition to the lost tumor suppression function, many mutated p53 (Mutp53) proteins acquire gain-of-function (GOF) activities as oncogenes to promote cancer progression, which manifest through aberrant expression of p53. In cancer cells with GOF Mutp53, statins can induce CHIP-mediated degradation of Mutp53 within the mevalonate pathway by blocking the interaction between mutp53 and DNAJA1. Therefore, targeting critical downstream pathways of Mutp53 provides an alternative strategy for treating cancers expressing Mutp53. In this review, we summarize recent advances with Wee1 inhibitors, statins, and mevalonate pathway inhibitors in cancers with p53 mutations.

## Introduction

Over 50% of all tumors harbor mutations in the p53 gene, demonstrating its criticality in tumor development (1). p53 is activated by a wide variety of stress signals to selectively transcribe a set of downstream target genes, thereby acting as a transcription factor to ultimately exert its tumor suppressive functions (1, 2). Many tumor hotspots result in mutated p53, not only with loss of tumor-suppressive functions, but also with a gain of new activities in promoting tumorigenesis, called mutant p53 gain-of-function (GOF) (1). Mutp53 not only promotes tumor development, but also renders these cancer cells with Mutp53 vulnerable to unique downstream pathways upon which they are dependent for survival as described in several reviews (3–5). The mutational status of p53 defines the efficacy of agents in mitigating the acceleration of multiple noteworthy cancers, of which has recently come to include Wee1 inhibitors and statins. Firstly, Wee1 is a nuclear protein essential for a cell cycle transition into mitosis: by catalyzing the inhibitory phosphorylation of a tyrosine residue on the Cdc2/cyclin B kinase complex, Wee1 negatively regulates mitotic entry by deactivating the complex's ability to regulate critical cell cycle proteins. Given Wee1's gatekeeper role in regulating mitotic progress, the potential consequences of Wee1 manipulation in p53 deficient cells requires further interrogation. Further, although the roles of p53 in regulation of cell cycle arrest, senescence and apoptosis have been widely discussed, the growing role of p53 in the regulation of ferroptosis and anti-oxidant defense is also critical for its tumor suppressive function (2, 3, 6, 7). p53 has demonstrated a repressive relationship on SLC7A11, a critical player in the cystine/glutamate antiporter; in downregulating this gene's expression and consequently promoting the non-apoptotic death of cells through ferroptosis (6, 8). Statins can inhibit the mevalonate pathway and promote degradation of mutp53 (9–11). Mevalonate-5-phosphate (MVP) promotes mutp53 stabilization by increasing the interaction between mutp53 and KSP40/DNAJA1 and inhibiting CHIP-mediated ubiquitination and proteasomal degradation of mutp53 (11). The mevalonate pathway inhibition can also sensitize cancer cells to ferroptosis by depleting CoQ10 and the biosynthesis of GPX4 by inhibiting tRNA isopentenylation via TRIT1 (12–16). In cancers with GOF Mutp53, statins can inhibit tumor growth by inducing mutp53 degradation, inhibiting prenylation of oncogenes and cholesterol synthesis, as well as inducing ferroptosis by inhibiting the biosynthesis of GPX4 and CoQ10 (16–18). The function of Wee1, as well as the progress of Wee1 inhibitors, statins and effect on immunotherapy in cancer with p53 mutations, will thus be discussed.

## Wee1 Inhibitors

**Figure 1:**
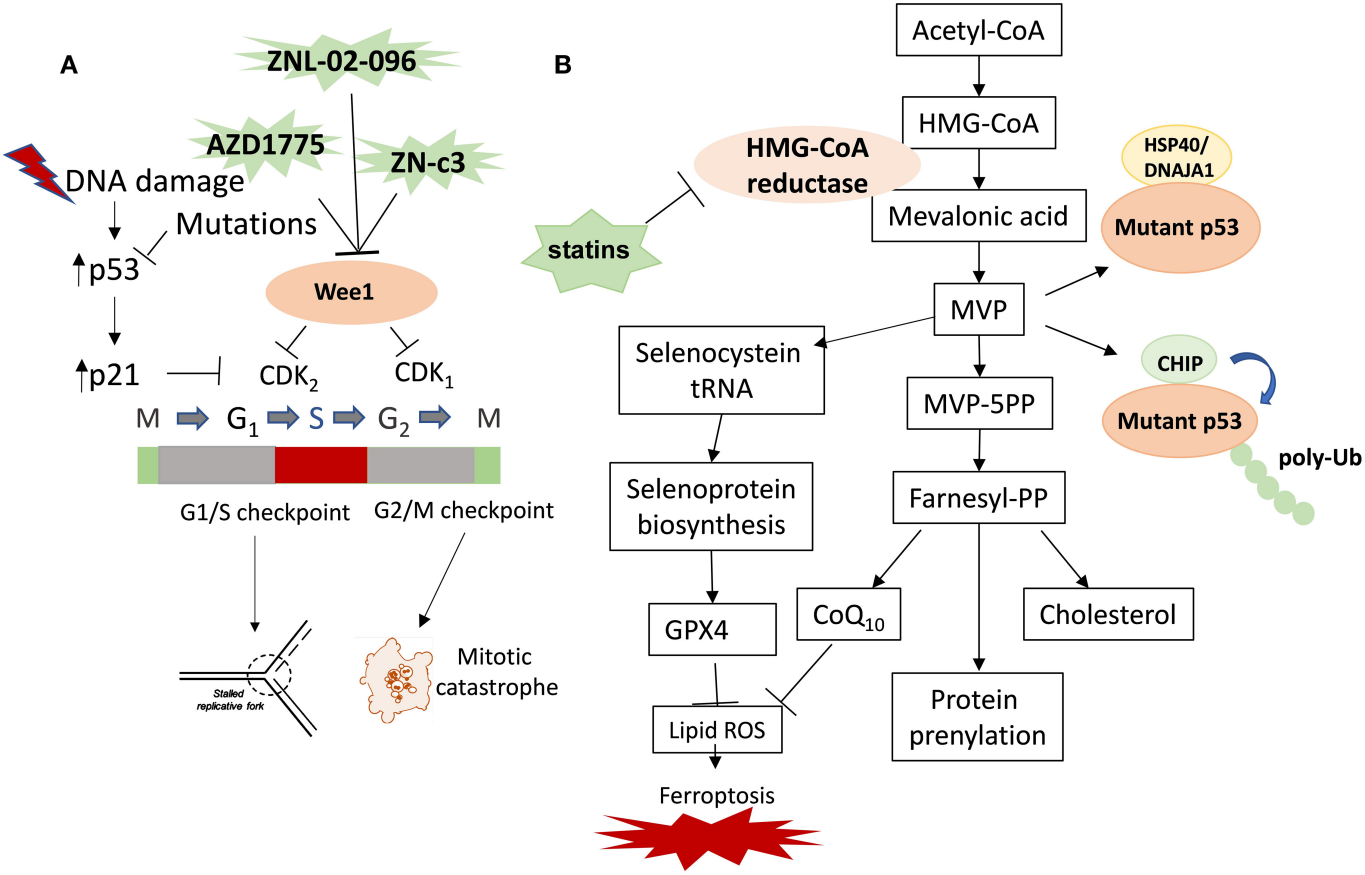
Therapeutic strategies targeting Wee1 and the mevalonate pathway in cancer with p53 mutations. The therapeutic strategies targeting mutp53 in cancer include targeting mutp53 directly or indirectly. The indirect strategies include inhibition of the critical downstream pathways of mutp53 and the direct strategies include inducing mutp53 degradation. **(A)** Wee1 inhibitor induces replication catastrophe and mitotic catastrophe in cancer cells with p53 mutations. **(B)** Statins inhibit mevalonate pathway and promote degradation of mutp53 and induce ferroptosis. Mevalonate-5-phosphate (MVP) promotes mutp53 stabilization by increasing the interaction between mutp53 and KSP40/DNAJA1 and inhibiting CHIP-mediated ubiquitination and proteasomal degradation of mutp53. Inhibition of the mevalonate pathway with statins represents a novel therapeutic strategy of targeting cancers with mutp53 by inducing mutp53 degradation, inhibiting prenylation of oncogenes and cholesterol synthesis, as well as inducing ferroptosis by inhibiting the biosynthesis of GPX4 and CoQ10.

In response to DNA damage, cell cycle checkpoints are activated to arrest cell cycle transition at the G1/S or G2/M by inducing the inhibitory phosphorylation on cyclin-dependent kinases Cdk1 and Cdk2, which is phosphorylated by the Wee1 kinase at tyrosine (Tyr)-15 and dephosphorylated by the Cdc25 phosphatases (19) (Figure 1). Both Wee1 and Cdc25 are precisely regulated by the fast-acting kinases-driven DNA damage response (DDR) network including PI3K family member DNA PK, ATM, ATR and downstream kinases Chk1 and Chk2 (20). Indeed, Wee1 is a nuclear protein essential for the regulation of G2 checkpoint passage: in catalyzing the Cdc2/cyclin B kinase complex at Tyr15, the protein is directly responsible for disabling this complex and arresting cells at this checkpoint, allowing for any detected DNA damage to be repaired rather than freely enabling entrance into mitosis (21). Furthermore, downstream genes regulated by tumor suppressor p53 play a critical role in cell cycle checkpoints at G1/S and G2/M in response to DNA damage to stop cell cycle transition and to prevent DNA replication collapse and mitotic catastrophe, a stage observed in cells prematurely entering mitosis that eventually promotes cell death caused by aberrant chromosomal segregation (1). Tumor cells lacking functional p53 are highly dependent on the G2/M checkpoint to maintain genomic stability, given that the mutation of p53 itself lends to diminished functionality of the G1/S checkpoint due to the loss of p53 target gene CDK inhibitor p21 transcriptional induction by p53 in response to DNA damage (22). Overriding the G2 checkpoint preferentially sensitizes p53-defected tumor cells to DNA-damaging agents and spares normal cells with wild type p53, which provides a potential therapeutic window for cancer cells with p53 defects, thereby leading to interest in proteins that directly oversee the G2 checkpoint (21, 23, 24). Given that mutp53 tumor cell growth is more likely to succumb to mitotic catastrophe if the G2 checkpoint and accumulated DNA damage is ignored, Wee1 inhibitors have posed as particularly intriguing therapeutic strategies towards achieving the aforementioned preferential sensitization of mutp53 cancer cells (23, 25). Three Wee1 inhibitors (Table 1) are currently explored in preclinical studies and clinical trials.

**Table 1:** Detailed information for Wee 1 inhibitors and Statins.

**Name(s)**	**Function**	**Pubchem CID/DrugBank accession number**	**Structure**
ZN-c3	Inhibitor of Wee1	131855165	C_2_H_4_O_6_Zn
AZD1775, MK-1775, Adavosertib	Inhibitor of Wee1	24856436/DB11740	C_27_H_32_N_8_O_2_
ZNL-02-096	Wee1 degrader		AZD1775 conjugated with CRBN-binding ligand
Atorvastatin	HMG-CoA^*^	60822/DB01076	C_33_H_35_FN_2_O_5_
Simvastatin	HMG-CoA^*^	54454/DB00641	C_25_H_38_O_5_
Lovastatin	HMG-CoA^*^	53232/DB00227	C_24_F_36_O_5_

* *HMG-CoA, hydroxymethylglutaryl-coenzyme A reductase*.

### AZD1775

The Wee1 inhibitor AZD1775 (also called adavosertib and MK1775) can override the G2/M checkpoint by activating cdc2, preventing the inactivating cdc2 phosphorylation at tyrosine 15 (Figure 1A). The single-agent activity of AZD1775/adavosertib was tested in paired tumor biopsies of patients carrying BRCA mutations as proof-of-mechanism to demonstrate the target modulation and DNA damage response at clinical trial NCT01748825 (11). AZD1775 was demonstrated to be safe and tolerable as a single agent and in combination with chemotherapy at the clinical trial NCT00648648 (Table 2) (8). The 21% response rate in TP53-mutated patients (*n* = 19) was higher than the 12% in TP53 wild-type patients (*n* = 33). Adavosertib monotherapy also demonstrated encouraging and durable evidence of activity in women with recurrent uterine serous carcinoma (USC). Liu et al. reported a phase II single-arm clinical trial (NCT03668340) of adavosertib in 34 patients with recurrent or persistent Uterine Serous Carcinoma (12). Median PFS was 6.1 months, and median duration of response was 9.0 months. Adverse events (AEs) included diarrhea (76.5%), fatigue (64.7%), nausea (61.8%), anemia (67.6%), low platelet count (17.6%), or low neutrophil count (32.4%) (13). Progression-free survival (PFS) was longer with adavosertib plus gemcitabine median 4·6 months vs. 3·0 months with placebo plus gemcitabine in platinum-resistant or platinum-refractory recurrent ovarian cancer patients at the phase 2 trial NCT02151292 (14). Adding adavosertib to chemotherapy improved PFS [median, 7.9 vs. 7.3 months] in patients with TP53-mutated, platinum- sensitive ovarian cancer treated with adavosertib (A + C) plus carboplatin and paclitaxel compared to placebo plus carboplatin and paclitaxel group (P + C) in the phase II clinical trial NCT01357161. Diminishing drug resistance to carboplatin via treatment with AZD1775 was observed in p53-mutant tumors resistant to first-line platinum-based therapy in a phase II clinical trial (8). An increase in adverse events of adavosertib was observed in patients treated with A + C vs. P + C: greatest for diarrhea (75; 37%), vomiting (63; 27%), anemia (53; 32%), and all grade ≥3 adverse events (78; 65%). Determining the quantity of adavosertib doses within the coupled chemotherapy treatment cycle or the potential for maintenance therapy through adavosertib should be considered to increase clinical benefit in future studies.

**Table 2:** Summary of listed clinical trials for Wee1 inhibitors and statins.

**Study**	**Design**	**Populations**	**Interventions**	**Clinical outcome**	**Conclusion and references**
NCT01748825	3 + 3 design, 10 ARMs with 2 expansion ARMs to determine maximum tolerated dosages (MTDs)	25 patients w/confirmed solid tumors unresponsive to all standard therapies; nine had BRCA1/2 mutations, with 6 enrolling in BRCA expansion cohorts	MK-1775 (AZD1775)	Common and dose-specific toxicities are GI-related/hematologic symptoms. MTD established at 225 mg BID for 2.5 days a week throughout a 21 day cycle. Partial responses in two patients with head/neck cancer and ovarian cancer, respectively.	AZD1775 single-agent activity in patients carrying BRCA mutations (26).
NCT00648648	Non-randomized, sequential assignment of open label AZD1775 monotherapy followed by lower doses/varied time schedules + chemotherapy to determine MTD w/chemo	Two hundred and two patients w/confirmed advanced tumors unresponsive to all standard therapy	MK-1775, Gemcitabine, Cisplatin, Carboplatin	Common toxicities include fatigue, nausea and vomiting, with maximum and biologically effective doses established for every treatment. Ninety four demonstrated stable disease, and 17 had partial responses.	Response rate higher in mutp53 patients than in wild-type. AZD1775 can be tolerable as a single agent and with chemotherapy (27).
NCT03668340	Non-randomized, parallel assignment of open label AZD1775 treatment (Phase II)	Thirty five participants w/confirmed serious uterine tumors presenting alongside p53 mutations, metastasis, measurable disease *via* biopsies	AZD1775	Overall response rate of 29.4%; 6-month PFS of 58.7%, median at 6.1 months with a median response duration of 9 months;	AZD1775 poses as a potential therapeutic method for uterine serous cancer
NCT02101775	Randomized, parallel assignment of single masked AZD1775 groups	Estimated 100 participants w/confirmed and measurable ovarian, peritoneal, or fallopian tube carcinoma; must be platinum resistant	Oral AZD1775/Adavosertib or placebo gemcitabine hydrochloride	NA	NA
NCT01357161	Randomized, parallel assignment of double masked AZD monotherapy v. paired chemo groups	One hundred and twenty one women with mutp53 and platinum sensitive confirmed ovarian cancer	MK-1775, Placebo, paclitaxel, carboplatin	Increased PFS of 7.9 vs. 7.3 months in AZD v. placebo groups, respectively; responsiveness varied based on p53 mutation subtype	AZD requires specific populations and regimens to be defined for most clinical benefit
NCT04158336	Single group assignment of open label treatment with ZN-c3 against solid tumors	39 treated subjects with advanced or metastatic tumors resistant to all current therapy models	Single dose escalation from 25 to 450mg orally QD	Of the five patients with stable disease and two with partial response, one PR had 42% reduced overall tumor burden, another with 56% reduction	ZN-c3 appears to be safe, tolerable, and therapeutically effective with a recommended Phase II dose of 300 mg, based on the MTD
NCT04516447	Non-randomized, parallel assignment of open label ZN-c3 treatment against solid ovarian tumors to determine the clinical activity and pharmacodynamics of ZN-c3 when coupled with carboplatin /PLD	Estimated 100 participants with platinum resistant high grade serious epithelial ovarian, fallopian tube, or peritoneal carcinomas	ZN-c3, Carboplatin, Pegylated liposomal doxorubicin	NA	NA
NCT04814108 (still in planning stages)	Phase II study to determine the safety and pharmacokinetics of ZN-c3 in uterine serous carcinoma; open label, single group assignment	Not yet selected	Zn-c3 as a single agent	NA	NA
NCT04767984	Randomized and parallel assignment, placebo-controlled targeting dominant-negative missense mutant p53 via Atorvastatin	Seventy participants with dominant-negative missense p53 mutations and are at risk of developing large intestinal cancer	Atorvastatin Calcium Placebo Administration	No results posted	N/A
NCT03560882	Single group assignment, pilot trial of atorvastatin in p53-mutant and p53-wild type malignancies	Fifty participants with conformational mutant tumor protein p53 (in solid tumor and relapsed acute myeloid leukemia)	Atorvastatin given 1–4 weeks at 80 mg/day	No results posted	N/A
NCT04491643	Single group assignment seeking to explore efficacy of megestrol acetate plus rosuvastatin for patients with early endometrial carcinoma	Forty participants who are women with early endometrial carcinoma	Megestrol Acetate Rosuvastatin	No results posted	Alternative treatment would be given to those who have stable disease after 6–8 months of treatment
NCT02767362	Single group assignment for evaluating anti-proliferative effects of atorvastatin on endometrium of endometrial cancer patients	Twenty four participants who are obese women that have endometrial cancer	Atorvastatin	No results posted	N/A
NCT04457089	Single group assignment for statin therapy to reduce progression of platinum sensitive ovarian cancer	Twenty participants with platinum-sensitive ovarian cancer	Simvastatin	No results posted	N/A
NCT00585052	Single group assignment for synergistic interaction of lovastatin and paclitaxel with ovarian cancer	Eleven participants with refractory or relapsed ovarian cancer	Paclitaxel Lovastatin	Ten of 11 patients completed study, all 10 had a progressive disease (PD), five of 10 experienced gastrointestinal disorders or hepatobiliary disorders	N/A

### AZD1775 Combined With Other Anticancer Agents

AZD1775 is not without flaw; namely, the drug has high toxicity at effective dosages, manifesting as the adverse side-effects as mentioned above. AZD1775 also tends to have lower than ideal specificity, notably targeting PLK1 alongside Wee1 in therapeutic trials. Given that Wee1 inhibition has been shown to have therapeutic efficacy, designs of more specific single-agents or modified combinations with lower toxicity at effective doses have been attempted. Firstly, Poly (ADP-Ribose) Polymerase (PARP) plays a critical role in maintaining genomic stability; as such, inhibition of PARP coincident with Wee1 inhibitors is theorized to synergistically increase the proportion of DNA-damaged cells prematurely passing through the checkpoint to become susceptible to mitotic catastrophe. Indeed, AZD1775 was observed to promote a synergistic cytotoxicity with the chemotherapeutic agent gemcitabine or the PARP1 inhibitor olaparib in p53-mutant ovarian and endometrial cancer cells (7). However, simultaneous induction of both treatments, although effective in inhibiting tumor growth, is offset by a strong toxicity and low tolerance characterized by weight loss and anemia by the end of a month-long treatment period. Fang et al. recently reported that the synergistic nature of PARP and Wee1 inhibitors was maintained while minimizing toxicity when sequential rather than concurrent therapies were employed in ovarian cancer xenograft and patient-derived xenograft models (10). These were significantly enhanced from the original monotherapeutic strategies and largely on par with concurrent outcomes to create reduced fork speed, increased DNA damage, and consequent slowed cell growth relative to normal cells based on endogenous replication stress levels and increased cell cycle arrest at the G1/S and G2/M checkpoints.

### ZNL-02-096

To increase Wee1 inhibition efficacy and specificity and decrease side effect of AZD1775, the Wee1 kinase degrader ZNL-02-096 was developed by binding AZD1775 to the cereblon (CRBN)-binding ligand pomalidomide by using PROTAC technique (28). Degradation upon conjugation of AZD1775 to pomalidomide functioned in a CRBN and proteasome-dependent manner, creating the novel and notably low-dose mechanism by which Wee1 overexpression in cancer cells may be repressed. ZNL-02-096 has recreated mono-therapeutic AZD- G2/M arrest rates at a significant 10-fold dosage decrease, thereby minimizing dosage-dependent toxicity while maintaining Wee1 inhibition efficacy and specificity. In particular, the secondary target PLK1 was avoided, indicating the reduction of blood-related side effects of this Wee1 degrader when used to treat patients in clinical trials.

### ZN-c3

Clinical activity of ZN-c3, another potent oral Wee1 inhibitor from Zentalis Pharmaceuticals, in a Phase 1 dose- escalation trial was recently reported in 55 patients with advanced or metastatic solid tumors at the AACR Annual Meeting 2021 (29) (Table 2). Better safety results were observed compared to adavosertib and partial responses in five patients were observed. The drug's safety profile could make it particularly well suited for use in combinations. Side effects were mostly mild to moderate, with nausea affecting about half of the 55 patients evaluable for safety, and diarrhea, fatigue and vomiting afflicting less than one-third of them. Of note, blood-related side effects struck <10% of patients: 1.8% of patients suffered a low white blood cell count, 7.2% of patients had a low platelet count and 7.2% of patients developed anemia. There are three clinical trials for ZN-c3 relevant to gynecological cancer, including a study of ZN-c3 in patients with solid tumors (NCT04158336), a study of ZN-c3 in patients with platinum-resistant ovarian cancer (NCT04516447) and a study of ZN-c3 in women with recurrent or persistent uterine serous carcinoma (NCT04814108). For the clinical trial in NCT04516447 in a study of ZN-c3 in patients with platinum-resistant ovarian cancer, levels of circulating deoxyribonucleic acid TP53 mutations tested by TAm-Seq will be correlated with response and changes in pCDC2 and pH2AX in skin and tumor tissue, which will be evaluated as pharmacodynamic markers of therapy. At clinical trial NCT04158336, ZN-c3 will be tested to treat patients with solid tumors with advanced or metastatic disease as a single agent and in combination with PARP inhibitor Talazoparib or PD1 inhibitor Pembrolizumab of immunotherapy.

### Wee1 Inhibitors Combined With Immunotherapy

Wee1 overexpression challenges immune cell killing (28, 30). This mechanism includes the protection of tumor cells from granzyme B/TNFα induced cell death. Activation of the G2/M cell cycle checkpoint was found to induce cell cycle arrest and protection from cell death by granzyme B and TNFα exposure, which was reversed with Wee1 kinase inhibitor AZD1775, leading to enhanced CTL killing of antigen-positive tumor cells and in bystander antigen-negative tumor cells of oral cavity carcinoma, melanoma and colon adenocarcinoma harboring variable Tp53 genomic alterations (19). Disruption of the G2/M cell cycle checkpoint in response to early and late CTL products can overcome the intrinsic resistance to CTL killing and provide rationale for the clinical combination of DDR inhibitors with immunotherapy (19, 31–34). In addition to combine with checkpoint inhibitors, combining inhibitor of Wee1 with ADC therapy was also studied (20). To sustain multiple myeloma (MM) plasma cell killing, DNA damage response (DDR) was triggered via phosphorylation of ATM/ATR kinases, CHK1/2, CDK1/2, and H2AX in MM cells by MEDI2228, a novel BCMA antibody-drug conjugate (ADC) delivering the DNA cross-linking PBD dimer tesirine in MM cells regardless of drug resistance and p53 status (20). PBD dimers are a class of DNA minor groove interstrand cross-linking (ICL) agents. Synthetic lethal was induced by combining MEDI2228 with DNA-damage repair (DDR) inhibitors in MM cells via modulation of RAD51 and accumulation of impaired DNA. Preclinical studies will further support ongoing clinical development of MEDI2228 combining with DDR inhibitors in patients with relapsed and refractory MM. Co-targeting of Wee1 and the DNA damage response kinase ATM was shown to downregulate PD-L1 expression in pancreatic cancer (15). Wee1 inhibition was found to sensitize cancer cells to immunotherapy via PD-1 checkpoint blockade in oral cavity carcinoma, melanoma and colon adenocarcinoma with variable Tp53 mutations, which provide a pre-clinical rationale for the combination of agents that target cell cycle checkpoints and activate anti-tumor immunity and simultaneously support the clinical trials of Wee1 inhibitor in combination with immunotherapy (16). The effect of the combination of Wee1 inhibitor ZN-c3 with PD1 inhibitor Pembrolizumab will also be studied in patients with solid tumors with advanced or metastatic disease at the clinical trial NCT04158336.

### Overexpression of SKP2 and CUL1 Predicts Benefit to Wee1 Inhibitors in Addition to p53 Defects

In addition to p53 mutations, overexpression of SKP2 and CUL1 in cancer patients may predict benefit to Wee1 inhibitors (25). Overexpression of G1/S regulatory genes, including SKP2, CUL1, and CDK2, was identified as resistance mechanisms to Wee1 inhibitors in a genome-wide unbiased genetic screen (25). Stable depletion of SKP2, CUL1, or CDK2 rescued sensitivity to Wee1 inhibition in breast and ovarian cancer cell lines, indicating that cancer patients with overexpression of these G1/S regulatory genes could respond robustly to Wee1 inhibitors (25).

## Mutant P53 and Mevalonate Pathway

Whereas Wee1 inhibitors specifically target loss of function p53 mutations, missense mutations in the TP53 gene can lead to the accumulation of dysfunctional TP53 (mutp53) proteins, which have gain-of-function activities that can activate SREPB transcription factors (specifically SREBP2) to upregulate mevalonate pathway enzymes (9). This, in turn, leads to tumor growth and progression. Further, this altered mutational state of p53 enables novel therapeutic methods distinct from those previously discussed. Dysfunctional TP53 proteins must first be stabilized to have tumor-promoting effects. The mevalonate pathway itself fulfills this role, bringing about a positive feedback cycle (18). The mevalonate pathway is critical in facilitating tumor proliferation as it produces necessary sterols and isoprenoids from acetyl CoA (19). These compounds, especially isoprenoids, are required for processes such as protein prenylation and lipidation that enable Ras and Rho GTPases to anchor to the cell membrane, both of which function in cell proliferation (18). Though there are many intermediates and reactions found within the mevalonate pathway, the action of HMG CoA reductase has been particularly of interest (9).

### GOF Mutp53 Activates Transcription by Interacting With SERBP2 and NF-Y

As previously mentioned, mutp53 stimulates the mevalonate pathway by binding to SREBP2, which is a transcription factor usually activated by normal p53. SREBP2 in turn affects cellular location and activates YAP and TAZ, both of which have previously been seen to function as potent oncogenes while also mediating the Hippo pathway, which prioritizes cell proliferation and survival (20, 21). In addition, mutp53 also interacts with nuclear factor Y (NF-Y), which functions to increase expression of the Rho family of small GTPases, which also function in cell proliferation. Interestingly, recent studies have provided evidence to believe that mutp53, through inducing SREBP2 and associated genes/pathways as well as NF-Y, can potentially transcriptionally activate HMG CoA reductase, thereby upregulating the mevalonate pathway as a whole and promoting survival pathways (20).

### Statins Interrupts Mutp53-DNAJA1

Luckily, it has been found that statins, a class of cholesterol- lowering drugs that have been used to prevent cardiovascular disease including atherosclerosis and coronary heart disease in people at high risk, inhibit HMG CoA reductase and therefore have a profound effect in inhibiting the mevalonate pathway in a variety of cancers including prostate cancer, non-small cell lung cancer, and ovarian cancer (18, 35). Additionally, these statins have minimal effects on WT p53 and DNA contact mutants, which offers the advantage of reducing side effects to normal cells with wild type p53 (Figure 1B). Statins accomplish this function by reducing mevalonate-5-phosphate (MVP), a metabolic intermediate in the mevalonate pathway (22). This triggers CHIP ubiquitin ligase-mediated degradation by disrupting the binding affinity between mutp53 and DNAJA1, a Hsp40 family member. Since mutp53 cannot bind or interact with DNAJA1, it will instead bind to CHIP and undergo degradation. The actual mechanism through which this interruption of mutp53-DNAJA1 interaction after reduction of MVP occurs is not entirely known but is hypothesized to be due to subsequent changes in protein folding machinery or post-translational modifications that affect DNAJA1 and/or mutp53 (22).

To further support the significance of DNAJA1 to mutp53 function, it was later found that knockout of DNAJA1 can also induce CHIP-mediated mutp53 degradation while overexpression antagonizes statin-induced mutp53 degradation (22). This latter effect is of particular interest because statin treatment is a method that is particularly effective against conformational mutp53 as statins inhibit mutp53 stabilization and protein prenylation, both of which are critical to mutp53's ability to carry out its effects. It was later determined that knockout of mevalonate kinase (MVK) has the same effect as reducing mevalonate 5-phosphate, suggesting that the disruption of mutp53 functions may be brought about through targeting and manipulating different parts of the mevalonate pathway (18). In addition to its effects on mevalonate 5-phosphate, statins inhibit HMG-CoA (HMGCR) reductase activity, which mediates the synthesis of cholesterol and the inhibition of the biosynthesis of selenoproteins (such as GPX4) and CoQ10, and thus enhance ferroptosis (11, 16, 17). Several groups have reported that Hsp90, Hsp40, CHIP and MDM2 play critical roles to stabilize mutant p53 *via* the HSP chaperone system, which suggests a possible synergism between HSP chaperone system inhibitors and statins in tandem (22, 24, 25, 30). Briefly, ferroptosis is a form of cell death that often occurs with iron accumulation and lipid peroxidation (36). It is often marked and caused by a decrease of intracellular glutathione and a decrease in glutathione peroxidase 4 (GPX4) activity. Also relevant is antioxidant coenzyme Q10 (CoQ10), the depletion of which sensitives cells to ferroptosis induced by FIN56, one of a few substances that can promote ferroptosis (36). In a study conducted in 2021, researchers took this information a step further and explored the effect of simvastatin used in conjunction with docetaxel, a chemotherapy drug, regarding ferroptosis in breast cancer. They found that simvastatin, as expected, inhibited GPX4 mRNA and protein expression and reduced tumor volume. As such, they concluded that simvastatin can, in fact, induce ferroptosis and sensitize cells to chemotherapy (37).

To support this previous information, it is worth mentioning that the gene signature of the activation of the mevalonate pathway was identified in an orthotopic model of epithelial ovarian cancer with the p53 mutation in 2016 (20). This model utilized aggressive abdominal ascites- derived 28-2 cells, which were found to have many upregulated genes of the mevalonate pathway. As expected, this upregulation had an association with the acquisition of the p53 mutation. Consistent with what was previously mentioned, treatment of these cells with a statin, simvastatin in this case, induced apoptosis in these cells via inhibition of HMG CoA reductase, the rate- limiting step of the mevalonate pathway that normally produces an intermediate that is converted into mevalonate 5-phosphate via mevalonate kinase (15). As an added note, it was found that 28- 2 cells were more sensitive than other parental cell lines (such as ID8) to statin treatment and that simvastatin-induced cell death could only be rescued by mevalonate and not by cholesterol, demonstrating the significance of interrupting the mevalonate pathway in dealing with mutp53 (20). This influence that statins, in general, possess over cell survival and death enables them to exercise their anti-tumor effects via influencing tumorigenesis related to oxidative stress, inflammation, and other means (15).

### Statins and Immunotherapy

Interestingly, there has also been discussion regarding combining statin therapy with immunotherapy (10, 38, 39). The immunotherapy drugs nivolumab, atezolizumab and pembrolizumab, function by blocking communication between programmed cell death-1 (PD-1) (40) and programmed cell death ligand-1 (PD-L1) have been shown to enhance the survival rates of those who subsequently receive statin treatment (10). This drug, along with others such as, as it was found that 10 patients who had statin treatment after immunotherapy had a much higher survival rate than those who did not receive statin treatment after immunotherapy. Statins was found to overcome resistance to PD-1 blockade therapies and improve the survival rate of KRAS mut tumor models of syngeneic colorectal cancer, genetically engineered lung and pancreatic tumors, indicating that KRAS mutation could be a molecular target for statins to elicit potent tumor-specific immunity (41). Statins can reduce inflammation by inhibiting isoprenoid synthesis of the mevalonate pathway (42). Statins also block inflammatory responses of endothelial cells and T cells by activating Kruppel-like transcription factors KLF2 and KLF4 (42). However, statins were also found to promote the differentiation of Forkhead box P3 (Foxp3+) CD4+ regulatory T cells (Tregs) cells while blocking the differentiation of proinflammatory helper T (Th17) cells (41–43). It is important to mention, though, that this finding is only observational and requires further testing to ensure its significance and validity (41–43).

### Statins in Clinical Trials of Cancer Patients

As of now, there are over 100 recorded clinical trials that implement statin treatment in cancer- related scenarios, including gynecological cancers. For instance, atorvastatin was used in the phase II cancer prevention clinical trial NCT04767984 in treating patients with ulcerative colitis who have dominant-negative missense p53 mutations and are at risk of developing large intestinal cancer (Table 2). Atorvastatin was also used in pilot trial NCT03560882, which will hopefully determine if atorvastatin given at a dose of 80 milligrams per day (mg/day) for 1 to 4 weeks can decrease the level of conformational mutant p53, Ki-67 and increase caspase-3 in patients with solid tumor and relapsed acute myeloid leukemia (AML). In gynecological cancers, there are several clinical trials involving statin treatment in ovarian cancer patients and endometrial cancer patients in which survival rates have been improved (15). The clinical trial NCT04491643 will explore the treatment efficacy of megestrol acetate 160 mg plus rosuvastatin 10 mg by mouth daily for 6 months in patients with early endometrial carcinoma (EEC) seeking for conservative treatment by hysteroscopy at every 3 months. The preoperative window, phase 0 study of trial NCT02767362 will evaluate anti-proliferative effects of atorvastatin for 2 to 4 weeks treatment by measuring Ki67 immunohistochemical staining in obese women who are to undergo surgical staging for endometrial cancer. The clinical trial NCT04457089, yet another example, is a single arm pilot trial to evaluate of the effect of simvastatin at 40 mg daily for ~6 months on cancer progression and change in serum level of CA125 among patients with platinum-sensitive ovarian cancer, treated with carboplatin and liposomal doxorubicin at Cedars-Sinai Medical Center. These platinum-sensitive ovarian cancer patients are at high risk of developing recurrent disease and have the potential to get the maximum benefit from simvastatin. Another clinical trial, NCT00585052, will evaluate if the treatment combination of paclitaxel and lovastatin is more effective for patients with refractory or relapsed ovarian cancer. Other than this, there are three recorded trials investigating the role of TP53 at statins associated with treatment to cancer. The clinical trial NCT02767362 will study the effect of atorvastatin for a minimum of 2 weeks with a dosage of 80 mg once daily on endometrial cancer. In clinical trial NCT03560882, atorvastatin will be used for a period of 1–4 weeks with a dosage of 80 mg per day to see efficiency and potency of atorvastatin and the effect on the reduction of mutant p53. In trial NCT04767984, atorvastatin will be investigated on its effect in reducing mutant p53 levels in patients. These trials demonstrate remarkable effort and drive to see if statins can be repurposed against the effects of mutp53 by blocking the mevalonate pathway in cancer patients with TP53 mutations.

### Concluding Remarks

As summarized above, Wee1 inhibitor and statins have shown to be effective to a certain extent and promising in both preclinical studies and clinical trials to cancer with p53 mutations; however, there are still unresolved obstacles. More studies on the application of Wee1 inhibitors and statins as monotherapy or combined with other reagents including immunotherapy in p53 mutated cancers will improve the therapeutic efficacy against p53 defect cancers.

## Author Contributions

XM generated the concepts for the topic and collected articles for review. Revisions and comments were coordinated by XM and SY. All authors participated in reviewing the collected literature, drafting the manuscript, and revising drafts.

## Funding

This manuscript was supported by NIH grants R01CA184101 (XM) and R37CA238274 (SY). XM was also supported by an Oberley award from HCCC. HCCC at the University of Iowa was supported by National Cancer Institute Award P30CA086862.

## Conflict of Interest

The authors declare that the research was conducted in the absence of any commercial or financial relationships that could be construed as a potential conflict of interest.

## Publisher's Note

All claims expressed in this article are solely those of the authors and do not necessarily represent those of their affiliated organizations, or those of the publisher, the editors and the reviewers. Any product that may be evaluated in this article, or claim that may be made by its manufacturer, is not guaranteed or endorsed by the publisher.
